# Initial experience of video-assisted thoracic surgery lobectomy after neoadjuvant chemotherapy plus toripalimab in a patient with locally advanced non-small cell lung cancer: a case report

**DOI:** 10.1186/s13019-021-01741-2

**Published:** 2022-01-16

**Authors:** Wei Li, Chunbo Zhai, Jianpeng Che, Weiqian Wang, Bingchun Liu

**Affiliations:** Department of Second Ward of Thoracic Surgery, The First Affiliated Hospital, Weifang Medical University (Weifang People’s Hospital), 151 Guangwen Street, Weifang, 261041 China

**Keywords:** Non-small cell lung cancer, Neoadjuvant immunotherapy, Video-assisted thoracic surgery, Toripalimab, Case report

## Abstract

**Background:**

Immune checkpoint inhibitors were used for patients with advanced non-small cell lung cancer (NSCLC) more and more frequently and the effects were thrilling. Toripalimab as a new immune checkpoint inhibitor has been shown to be effective in patients with advanced NSCLC. However, data regarding the safety and feasibility of surgical resection after treatment with toripalimab for NSCLC remain scarce. Here, we present a case with locally advanced NSCLC that received video-assisted thoracic surgery (VATS) lobectomy after treatment with toripalimab in combination with chemotherapy.

**Case presentation:**

A 62-year-old male patient with a history of coronary artery stenting operation for two times was found a 3.4 × 3.2 cm cavity mass in the upper lobe of the left lung and enlarged left hilar and mediastinal lymph nodes. Pathological results identified squamous cell carcinoma. The patient was diagnosed with a locally advanced NSCLC and received VATS left upper lobectomy and lymph node dissection after neoadjuvant chemotherapy plus toripalimab for 3 cycles. The postoperative pathological results showed complete tumor remission. Short-term follow-up results were excellent, and long-term results remain to be revealed.

**Conclusions:**

Our preliminary results showed that the use of neoadjuvant toripalimab and chemotherapy for the locally advanced NSCLC before surgical resection is safe and feasible.

## Background

Toripalimab as a humanized immunoglobulin G4 monoclonal antibody targeting programmed cell death protein 1 (PD-1) was approved for the treatment of unresectable or metastatic melanoma after the failure of previous systemic therapy in China on December 17th, 2018 [1]. A phase 1 trial characterized the encouraging antitumor activity and a controllable safety profile of toripalimab in patients with advanced NSCLC [2]. However, data regarding the safety and feasibility of surgical resection after treatment with toripalimab in combination with neoadjuvant chemotherapy remain scarce. We recently completed a VATS lobectomy for a patient with locally advanced NSCLC after treatment with toripalimab in combination with docetaxel and cisplatin. We expect that this case report will provide initial insights into the efficacy, safety, and impact of toripalimab on surgery.

## Case presentation

A 62-year-old male with a chief complaint of bloating visited our hospital on May 29^th^, 2020. The patient with an 80 pack-year history of smoking underwent coronary artery stenting operation two times (One was on August 10th, 2017, the other one was on October 1st of 2017) due to coronary atherosclerotic heart disease. This patient has been treated with Plavix, aspirin, rosuvastatin and isosorbide mononitrate.

**Fig. 1:**
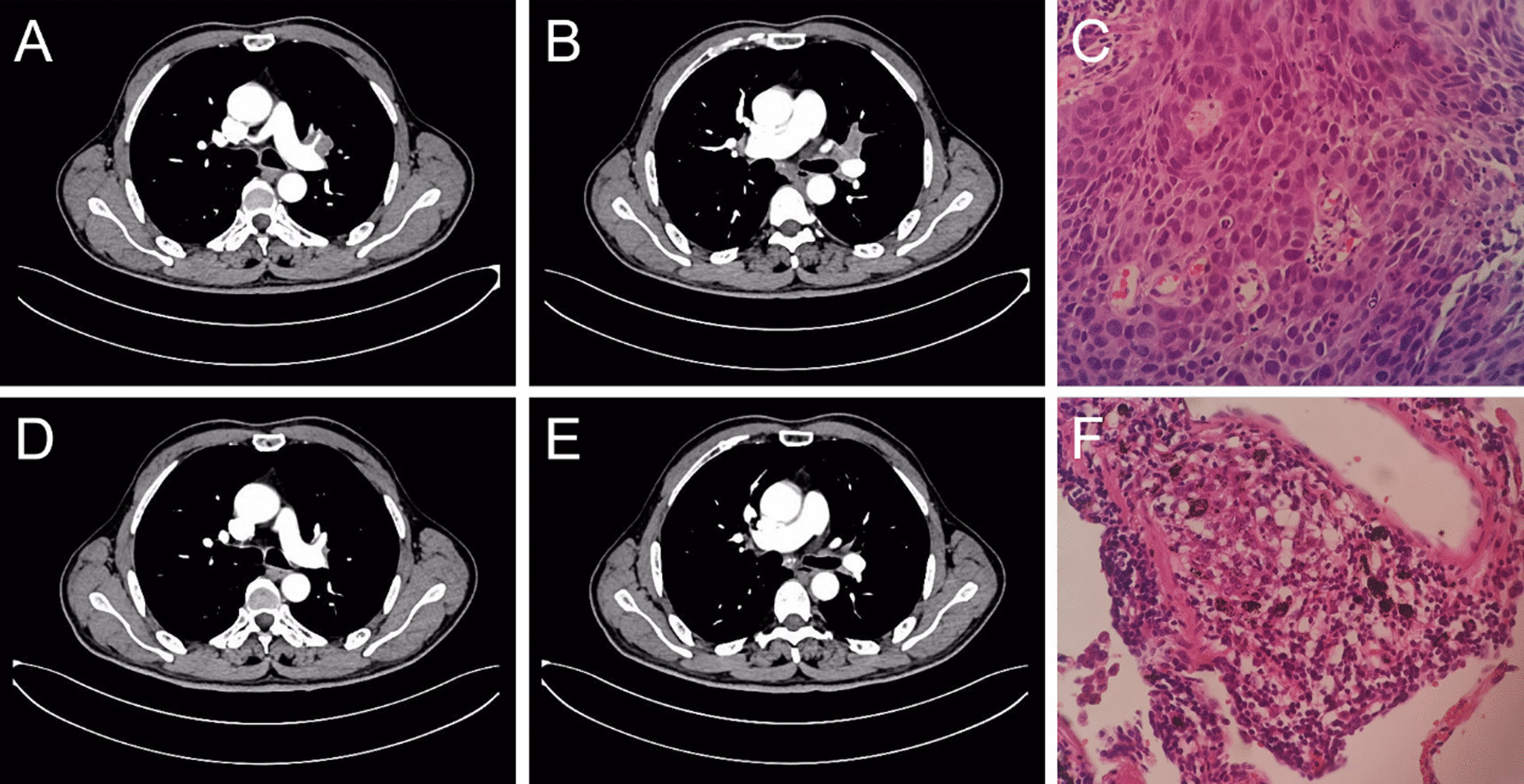
**A** and **B** are CT images before the treatments of neoadjuvant chemotherapy plus toripalimab. **C** is pathological image before the treatments. **D** and **E** are CT images after 3 cycles’ treatments of neoadjuvant chemotherapy plus toripalimab. **F** is pathological image after the treatments of neoadjuvant chemotherapy plus toripalimab followed by VATS lobectomy

During physical examination, no obvious abnormal breath sounds in the chest or abnormal cervical lymph nodes were found. An enhanced computed tomography (CT) of the chest and positron emission tomography-computed tomography (PET-CT) showed a 3.4 × 3.2 cm cavity mass in the upper lobe of the left lung and enlarged left hilar and mediastinal lymph nodes, with the largest node being about 2.6 × 2.8 cm (Fig. 1A,B). There were no obvious metastases based on magnetic resonance imaging of the brain and PET- CT of the abdomen and bone. Analysis using a fiber optic bronchoscope identified a tumor which blocked the opening of the proper segment of the left upper lobe. The pathological result by biopsy confirmed squamous cell carcinoma (Fig. 1C).

Measurements of serum tumor markers showed elevated level of cytokeratin 19 fragment (Cyfra21-1: 4.77 ng/mL, reference: ≤ 3.3 ng/mL), neuron-specific enolase (NSE: 19.60 ng/mL, reference: ≤ 15.2 ng/mL), and squamous cell carcinoma antigen (SCCA: 2.58 ng/mL, reference: ≤ 2.5 ng/mL). The results were negative for serum carbohydrate antigen 125 (CA125), carcinoembryonic antigen (CEA) and vascular endothelial growth factor (VEGF). Immunohistochemical staining showed positive staining for CK5/6, P40 and PD-L1 (+ 80% tumor cells), and negative staining for TTF-1, PD-1 and weakly positive staining for ALK-Ventana. Molecular analysis indicated no *EGFR* mutation or *ROS1* fusion.

Combined with the patient’s chest enhanced CT, bronchoscopy, and PET-CT conditions, the patient was diagnosed with a locally advanced NSCLC with TNM stage of cT4N1M0 stage IIIA. On one hand, left pneumonectomy may be necessary if direct surgery was performed. However, the patient has a history of two times’ coronary artery stenting operations which made the surgical risk of left pneumonectomy very high. On the other hand, it was very likely that lobectomy alone may not achieve complete microscopic resection (R0). It was therefore recommended to give neoadjuvant chemotherapy plus toripalimab for 3 cycles after lung cancer multiple-disciplinary team (MDT) assessment. If the tumor and lymph nodes are significantly reduced, then surgery will be given. If the tumor does not shrink, radiotherapy and chemotherapy will be given. The patient agreed with the treatment plan and signed an informed consent form.

From June 25th, 2020 to August 6th, 2020, the patient accepted 3 cycles’ neoadjuvant chemotherapy (docetaxel 130 mg and cisplatin 130 mg) plus immunotherapy (toripalimab 240 mg). the patient’s chest enhanced CT on August 5th, 2020 showed that the tumor remission was significant(Fig. 1D, E). After an MDT evaluation, surgical treatment was recommended after the third cycle’s treatment. The patient's perioperative physical conditions and surgical indications were evaluated by enhanced CT of brain, chest and abdomen, and by coronary CT angiography, pulmonary function, and echocardiography.

**Fig. 2:**
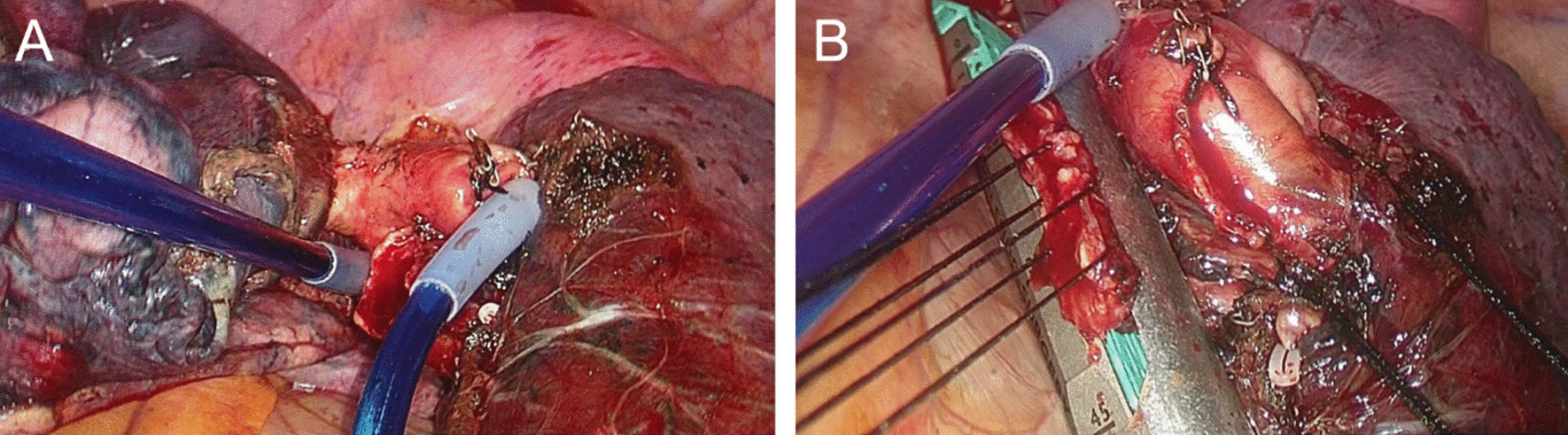
In **A**, vascular blocking tapes were put on the left pulmonary artery root and lower pulmonary artery respectively. In **B**, the upper lobe bronchus stump was suspended with 3 silk threads and closed with an endoscopic linear cutting stapler

The patient received the VATS left upper lobectomy and lymph node dissection on September 16th, 2020. The procedure was as follows: The thoracoscopic observation hole was at the 7th intercostal space of the mid-axillary line, and the operative hole was at the 4th intercostal space of the anterior axillary line and the 7th intercostal space of the posterior axillary line. The branches of the lingual segmental artery, posterior segmental artery and left upper pulmonary vein were cut respectively by endoscopic linear cutting stapler after opening the fissure. Anterior apical artery and left superior lobar bronchus were densely connected by fibrotic lymph nodes. The left superior lobar bronchus was firstly cut with scissors at the bifurcation of lingual bronchus and proper bronchus. However, the adhesion of lymph nodes and blood vessels was still very dense. Thus vascular blocking tapes were put on the left pulmonary artery root and lower pulmonary artery respectively to avoid hemorrhage during operation (Fig. 2A). The lymph nodes were carefully separated with scissors from the blood vessels after the vessels were blocked. The anterior apical artery was then cut with an endoscopic linear cutting stapler. The tumor and lung were placed in retrieval bags and removed through the front operation hole. Then the upper lobe bronchus stump was suspended with 3 silk threads and closed with an endoscopic linear cutting stapler (Fig. 2B). Intraoperative frozen pathological examination showed that there was no residual tumor at the cut edge of the bronchus. Finally, the lymph nodes in groups 4, 5, 6, 7, 9, 10, and 11 were completely removed. Intraoperative blood loss was about 50 ml without blood transfusion.

In the perioperative period, the patient’s vital signs were stable, and there were no complications such as arrhythmia, thrombosis, pulmonary infection, and air leakage. The postoperative routine pathological results showed that the tumor had achieved complete remission (Fig. 1F). The patient recovered well and was discharged on the 7th day after surgery. Moreover, the patient received the 4th cycle’s chemotherapy and immunotherapy consistent with preoperative treatment plan on October 14th, 2020.

## Discussion and conclusions

Previous study showed that patients with locally advanced (T2-T4) NSCLC or limited lymph nodal metastasis (N1, unilevel N2) need a multimodal therapy including surgery combined with adjuvant cisplatin duplet chemotherapy. However, complete microscopic resection (R0) and adjuvant chemotherapy only improves survival benefit by approximately 5% [3, 4]. In recent years, preoperative neoadjuvant chemotherapy combined with immunotherapy has achieved surprisingly high remission rates. The results of Checkmate159 trial showed that the major pathological response (MPR) of the nivolumab was 45% [5]. The results of NCT02716038 showed that the MPR rate of atezolizumab combined with platinum-based dual regimen was up to 57% [6]. The patient in our study was a locally advanced case because of the significantly enlarged and metastatic hilar lymph nodes. In this case, complete microscopic resection (R0) may not be achieved, and the long-term survival benefit may also be unsatisfied if operation was performed directly. Thus neoadjuvant chemotherapy plus immunotherapy for this patient was recommended by MDT assessment.

Toripalimab has been used to treat several kinds of unresectable or metastatic malignant tumors and the application of toripalimab in advanced NSCLC are increasingly reported. Preliminary data suggested that its safety and effectiveness were encouraging [7–10]. In particular, a recent single-center, open-label, parallel-control, phase 1 trial (NCT03301688) demonstrated the high security and exciting antitumor activity of toripalimab in patients with advanced NSCLC [2]. The trial results suggested that patients with PD-L1 expression of more than 50% could probably achieve long-term survival benefits and lasting response. In this case report, we presented some initial results about the surgical outcomes of VATS lobectomy after combined application of toripalimab with chemotherapy. Our preliminary results showed that there was a good pathological response and no apparent adverse events observed in the case [11–13]. The use of neoadjuvant chemotherapy and toripalimab for the locally advanced NSCLC before surgical resection was found to be safe and feasible. Our results suggest that toripalimab might be a promising treatment option for locally advanced lung cancer patients who have high expression of PD-L1. More studies are needed to further explore the potential role or effects of toripalimab in neoadjuvant combinations for resectable NSCLC.

Previous studies have shown that some patients still have residual tumors in radiology after receiving immunotherapy despite that the pathological remission has actually occurred [12, 14]. Similarly, in this case, the preoperative enhanced CT showed that there are still a small number of residual tumors and lymph nodes after 2 cycles’ neoadjuvant toripalimab immunotherapy in combination with cisplatin duplet chemotherapy. However, the postoperative pathological results showed that the tumor and lymph nodes achieved complete remission. These results suggested that traditional response criteria used to evaluate the effects of cytotoxic chemotherapy may be not suitable for checkpoint inhibitors [15]. Therefore, surgical resection is still an important option for the patients with residual tumors and/or lymph nodes in radiology.

Dense fibrosis as a result of an excellent response to immunotherapy may increase the difficulty of surgery and even lead to conversion to thoracotomy from thoracoscopy [12]. During the operation, it was found that the residual tumor tissues, lymph nodes, upper lobe bronchus and pulmonary artery of this patient were densely adhered, and the normal interstitial space disappeared. In this case, it was difficult to complete the operation with conventional thoracoscopic techniques. To solve this issue, a novel strategy was designed: the bronchus was cut with scissors at the bifurcation of the upper lobe lingual and proper bronchus after the left upper pulmonary vein and branches of pulmonary artery were treated first. Thus the relationship between the lymph nodes and the pulmonary artery can be better exposed, Scissors were then used to push the lymph nodes to the distal end of the pulmonary artery after the blocking tape temporarily blocked the left pulmonary artery stem and the lower pulmonary artery. Finally, the bronchial stump was closed with an endoscopic linear cutting suture device. The advantage of this strategy was that the tumor and lymph node can be fully assessed under the premise of ensuring safety. The relationship between trachea and pulmonary artery not only achieves the purpose of R0 resection, but also avoids hemorrhage and transfer to thoracotomy. Therefore it is a better method for VATS lung resection after immunotherapy.

Some limitations were unavoidable in this study. First, only one case was reported in this study. Our conclusions need to be further validated with more patient samples in the future. Second, since the main purpose of this study was to investigate the effect of toripalimab on perioperative period, long-term follow-up of patients was insufficient, and long-term follow-up of patients receiving toripalimab will be needed in future studies to assess overall survival and disease-free survival. In addition, lots of questions remain to be answered, such as how to determine preoperative complete remission of the tumors and whether surgery is necessary for these complete remission patients.

In conclusion, this case report suggested that toripalimab may be a promising choice for locally advanced NSCLC patients. Although surgery is challenging after immunotherapy, for the patients with resectable opportunity following immunotherapy, the VATS operation may deserve a try after temporarily blocking the pulmonary blood vessels to ensure the safety in operation.

## Data Availability

Not applicable.

## Abbreviations

NSCLC: Non-small cell lung cancer
VATS: Video-assisted thoracic surgery
PD-1: Programmed cell death protein 1
CT: Computed tomography
PET-CT: Positron emission tomography-computed tomography
CA125: Carbohydrate antigen 125
CEA: Carcinoembryonic antigen
VEGF: Vascular endothelial growth factor
MDT: Multiple-disciplinary team
MPR: Major pathological response
